# PReS-FINAL-2338: Fate of lymphocytes after withdrawal of Tofacitinib treatment

**DOI:** 10.1186/1546-0096-11-S2-P328

**Published:** 2013-12-05

**Authors:** A Tommasini, E Piscianz, E Valencic, E Cuzzoni, G Decorti, A Taddio

**Affiliations:** 1 Department of Pediatrics, Trieste, Italy; 2 Institute for Maternal and Child Health IRCCS Burlo Garofolo, Trieste, Italy; 3 Università di Trieste, Trieste, Italy

## Introduction

Tofacitinib (Tofa) is an inhibitor of Janus Kinase 3, developed for the treatment of autoimmune diseases and for the prevention of transplant rejection. Thanks to its selective action on proliferating cells, Tofa can offer a way to block T cell activation. The potential field of clinical application is thus represented by disorders with inappropriate T cell response, such as rheumatoid arthritis, psoriasis, graft rejection, ulcerative colitis and graft versus host disease. However, the tuning of efficacy (suppression of pathogenic lymphocytes) and safety (suppression of protective immunity) remains an open issue.

Unexpectedly, whereas the drug has been widely used in animal models and has been already introduced into the clinics, only few studies had investigated its immunological potential *in vitro*.

## Objectives

To study the effect of tofacitinib in vitro stimulated lymphocytes. In particular, to measure the effect of the drug on cell proliferation, lymphocyte subsets and cell viability during treatment and after interruption of treatment.

## Methods

Healthy donors' peripheral blood mononuclear cells are stimulated or not with phytohemoagglutinin and incubated for 4-days with different concentrations of tofacitinib. After the first incubation, cells are washed twice and further incubated for 4-days without stimuli and drug. Cell proliferation is assessed by CFSE dilution assay; cell viability by 7-AAD staining; lymphocyte subsets are analyzed by multicolour flow cytometry.

## Results

Here we showed that Tofa exerts a rapid and strong effect on lymphocyte activation, leading to a complete arrest in proliferation and to a strong down-regulation of activation markers in PHA stimulated lymphocytes. Notably, these results are achieved with a negligible toxicity on lymphocyte viability. However, after the withdrawal of the drug, stimulated lymphocytes resume proliferation. Thus, transient treatment with Tofa didn't lead to a relevant inhibition of final proliferation, but it strongly affected the distribution of lymphocyte subsets, with a reduction of NK cells, B cells and CD8 cells.

## Conclusion

Based on these data, we can presume that discontinuation of the drug after a short treatment may lead reactivation of diseased lymphocytes and to a reduction of NK and B cells as well *in vivo*, possibly resulting in undesired effects of the drug. To evaluate this possibility, a careful study of the expression of lymphocyte activation markers and of the distribution of lymphocyte subsets should be performed in all subjects after discontinuation of Tofa treatment.

## Disclosure of interest

None declared.

